# Prevalence of syphilis among female sex workers and their clients in Togo in 2011

**DOI:** 10.1186/s12889-017-4134-x

**Published:** 2017-02-21

**Authors:** Wemboo Afiwa Halatoko, Dadja Essoya Landoh, Bayaki Saka, Koffi Akolly, Yao Layibo, Issifou Yaya, Dodji Gbetoglo, Abiba Kere Banla, Palokinam Pitché

**Affiliations:** 1 Institut National d’Hygiène de Lomé, Lomé, BP 1396 Togo; 2 World Health Organization, country office of Togo, Lomé, Togo; 3 Service de Dermatologie et IST, CHU Sylvanus Olympio, Université de Lomé, Lomé, Togo; 4 Laboratoire de Santé Publique (EA 3279), Aix-Marseille Université, Marseille, France; 5 Unité de Recherche Démographique, Université de Lomé, Lomé, Togo; 6 Conseil National de lutte contre le VIH/Sida et les IST (CNLS/IST), Lomé, Togo

**Keywords:** Female sex workers, Clients, Syphilis, HIV, Togo

## Abstract

**Background:**

During the last ten years, a resurgence of syphilis has occurred in many countries worldwide, including Togo. Previous studies have shown a wide range of syphilis infection among the female sex workers (FSWs), from 1.5 to 42.1%. In Togo, Key populations, including FSWs, are rarely involved in the sentinel surveillance programs to determine the prevalence of HIV and syphilis. The aim of this study was to determine the prevalence of syphilis among female sex workers (FSWs) and their clients in Togo.

**Methods:**

We conducted a cross-sectional study in December 2011 targeting FSWs and their clients in Togo. Among participant who consented, we collected blood samples for syphilis and HIV testing.

**Results:**

In total, 1,836 participants (1,106 FSWs and 730 clients) were included in the survey. Their mean age was 28.6 ± 9 years. The prevalence of syphilis was 2.2% (2.2% among FSWs compare to 2.3% among their clients, *p* = 0.82). This prevalence was higher among FSWs over 30 years old compare to those less than 30 years old (Odd Ratio (OR) =5.03; 95% CI [1.95-13.49]). Single FSWs were three times less likely to have syphilis than those living in couple or married (OR = 3.11; CI 95% [1.16-8.83]). Brothel based or declared FSWs were 4 times more likely to be infected by syphilis than secret ones (OR = 3.89; CI 95% [1.60-9.54]). Out of the 1,836 participants of the survey, 165 (8.9%) were HIV positive. Having syphilis was associated with HIV infection (OR = 3.41; IC 95% [1.53-7.41]).

**Conclusion:**

This study showed that: i) the prevalence of syphilis among FSWs and their clients was high; ii) syphilis was significantly associated with HIV infection. It is necessary to increase awareness campaigns and emphasize on condom use among this key population group.

## Background

During the last ten years, a resurgence of syphilis has occurred in many countries worldwide including Togo, making the prevention of syphilis one of the priorities of national programs established to control STI/HIV/AIDS [1–4]. Each year, the World Health Organization (WHO) estimates that about 12 million new cases of syphilis occur in the world of which more than half are recorded in sub-Saharan Africa [5]. In this region, several studies have shown that the prevalence of syphilis is higher among groups with risky sexual behaviour such as sex workers, drug users and Men who have Sex with Men [6–10]. Previous studies have shown a wide range of syphilis infection prevalence among the female sex workers (FSWs), from 1.5 to 42.1% [8–10]. These studies also reported that the risk of contracting syphilis was influenced not only by the socio-demographic profile and sexual behaviour of FSWs [8–13], but also by the type of sex work [11].

Data collection through surveillance of STIs in individuals with higher risky sexual behaviour such as FSWs is recommended to monitor HIV epidemic [14].

In Togo, data on the prevalence of syphilis are obtained during sentinel surveillance among pregnant women attending antenatal clinics to monitor the trends of STIs prevalence. In 2010, the prevalence of syphilis was 1.2% [15]. Key populations including FSWs are rarely involved in these surveillance programs. In 2011, a second-generation surveillance of HIV and syphilis was conducted at the national level to determine the prevalence of HIV and syphilis among FSWs and their clients. The objective of this study was to determine the prevalence of syphilis among FSWs and their clients in Togo in 2011.

## Methods

### Study design

We conducted a cross-sectional study between 17 and 27 December 2011 among FSWs and their clients in the whole country.

### Population and sampling

This study targeted FSWs and their clients. Male sex work in Togo is very limited [16]. A FSW was defined as any woman who exchange sexual favours for money or goods either in brothels (declared FSW) or in hot spots such as bars, night clubs, hotels or on streets (street-based or secret FSW) during the past 6 months. Any male individual who visited sex work sites (brothels, hot spots) at least one time to satisfy his sexual desire with a FSW was considered to be a client. A national mapping of FSWs was conducted in 2009 to determine the number of FSWs by region and identify the different sex work sites [17]. This study reported 8,000 FSWs from whom 490 were declared FSWs, 1,548 street-based and 5,962 secret ones [17]. These results were used as a frame for sampling FSWs in each health district of Togo. We sampled at least 15% of the total FSWs. In total 1,200 FSWs were estimated to be included in the survey. On this basis, it was planned to interview 490 declared FSWs who could be identified in 2011 because of the easy access to this group compared to other categories of FSWs. For the remaining 710 FSWs, the sampling was carried out among the street-based and secret FSWs in ratio to their weight; 19% for street-based FSWs and 75% of secret FSWs. For our study, we considered two categories of sex workers: i) Brothel-based who were defined as declared FSWs and ii) Covert and street-based FSWs.

During data collection, all the FSWs or their clients who were present and who gave their consent were included in the survey.

### Data collection

The study was carried out using the second generation of the generic surveillance protocol developed by WHO and UNAIDS [14] and was validated by the national reference group for monitoring, evaluation and research on AIDS and STIs. The second generation surveillance protocol helped countries develop systems to collect information on HIV trends to monitor their epidemics and to gather information to improve prevention, strategies, planning and evaluation [14, 18]. We trained the investigation team and field workers on the use of data collection tools [16]. We used a semi-structured questionnaire for data collection. We collected data on the following variables: city of residence, sex work site, educational level, age, sex, occupation, use of condoms, number of sexual partners, syphilis and HIV serology results.

We collected blood samples from consenting participants for syphilis and HIV tests. We centrifuged samples, aliquoted and stored them at −20 °C for a maximum of one week before their conveyance with cold chain to the National Institute of Hygiene of Lomé. We used hemagglutination test (TPHA) and an immunochromatographic treponemic test: SD Bioline rapid tests to diagnose syphilis. We used, ELISA and rapid tests to perform HIV testing. These tests were designed to detect antibodies (IgG, IgM, IgA) of *Treponema pallidum* and antibodies of HIV-1 (p24, gp41) and HIV-2 (gp36) in serum. We considered a participant to be syphilis-positive if the rapid test and TPHA were simultaneously positive and a subject to be HIV-positive if ELISA and the rapid test were simultaneously positive.

### Data analysis

We used Epi-Info 3.5.1 to analyse data. We calculated frequencies and performed bivariate analysis using chi square test or Fisher’s exact test when appropriate at a 5% significance level. We calculated Odd Ratios (OR) and their 95% confidence intervals (CI).

### Ethical considerations

We explained clearly to each participant the objectives and the benefits of this study as well as their right to interrupt the interview without any justification. Participants diagnosed positive for HIV or syphilis were referred immediately to the HIV clinic for treatment and support. We obtained a clear written informed consent from each study participant. We ensured confidentiality and anonymity of participants. The names of participants in the survey were replaced by numbers to ensure confidentiality.

## Results

### Socio-demographic and behavioural data

During the survey, 1,836 people participated including 1,106 FSWs and 730 clients. Out of them, 858 (46.7%) were in the Lomé region and 325 (17.7%) were in the Savana region (Table 1). Of the 1,106 FSWs, 73.4% were covert or street-based and 26.6% were brothel-based. The mean age was 27.6 years (range: 13 to 68 years) for FSWs and 32.5 for clients (range: 22 to 76 years).

**Table 1:** Prevalence of Syphilis and Associated Factors among FSWs and their Clients in Togo in 2011

	Syphilis positive n (%)	Total	OR CI 95%	p
Health regions				
Lomé commune	27 (3.1)	858	-	0.09
Maritime	0 (0.0)	154		
Plateaux	3 (1.0)	287		
Centrale	2 (3.1)	65		
Kara	2 (1.4)	147		
Savane	7 (2.2)	325		
Sex				
Male (Client)	17 (2.3)	730	1.07 [0.55-2.10]	0.82
Female (Sex Workers)	24 (2.2)	1106		
Age in years (All)				
≥ 30	25 (3.6)	686	2.68 [1.37-5.30]	*0.001*
< 30 years	16 (1.4)	1150		
Age in years (FSWs)				
30 and over	17 (4.6)	369	5.03 [1.95-13.49]	*<0.001*
< 30	7 (1)	736		
Age in years (Clients)				
≥ 30	8 (2.4)	327	1.10 [0.38-3.14]	0.84
< 30	9 (2.2)	403		
Marital status (All)				
Married/in couple	27 (2.9)	929	1.91 [0.96-3.86]	0.05
Single	14 (1.5)	907		
Marital status (FSW)				
Married/in couple	18 (3.3)	549	3.11 [1.16-8.83]	*0.012*
Single	6 (1.1)	557		
Marital status (Clients)				
Married/in couple	9 (2.4)	379	1.04 [0.39-2.73]	0.93
Single	8 (2.3)	351		
Sex Work sites				
Brothel-based	14 (4.8)	294	3.89 [1.60-9.54]	*<0.001*
Covert and street-based	10 (1.3)	788		
HIV serology				
HIV positive	10 (6.1)	165	3.41 [1.53-7.41]	*0.001*
HIV negative	31 (1.9)	1670		

*Note*: *FSWs* Female Sex Workers

*HIV* Human Immuno deficiency Virus

*OR* Odd ratio

The significant p values are in italic

We found that, 409 (37%) out of the 1,106 FSWs had their first sexual intercourse before the age of 18 years old, and over 43% of FSWs had more than seven clients per week. Of the 1,106 FSWs, 88% reported using a condom during their last sexual intercourse before the survey and 61% (445/730) of the clients reported the same.

### Prevalence of syphilis and HIV

Of the 1,836 participants, 41 (2.2%) were infected with syphilis. There was no significant difference between the prevalence among FSWs and that of their clients (2.2% vs. 2.3%; *p* = 0.82). Even though the difference was not significant (*p* = 0.0.09) (Table 1), the prevalence of syphilis was higher in the central region (3.1%) and Lomé district (3.1%) than in other regions. Participants aged more than 30 years of age were more likely to be infected than those aged less than 30 years (OR = 2.68; 95% CI [1.37-5.30]). Among FSWs, those aged more than 30 years were 5 times more likely to be infected than those aged less than 30 years (OR = 5.03; 95% CI [1.95-13.49]). Declared or brothel-based FSWs were 4 times more likely to be infected than secret ones (OR = 3.89; CI 95% [1.60-9.54]). Single FSWs were three times less likely to have syphilis than married FSWs or those living in couple (OR = 3.11; CI 95% [1.16-8.83]).

Out of the 1,836 participants, 165 (8.9%) were tested HIV positive. HIV seroprevalence was 13.1% among FSWs against 2.7% among clients. Among brothel-based FSWs, 64/294 (21.8%) were HIV positive compared to covert and street-based FSWs 82/812 (10.1%). Having syphilis was associated with HIV infection (OR = 3.41; CI 95% [1.53-7.41]).

## Discussion

We determined the prevalence of syphilis and identified some factors associated with syphilis infection among FSWs and their clients in Togo. We found that: i) the prevalence of syphilis among FSWs and their clients was two times higher than estimates reported in a previous study among pregnant women attending antenatal clinics [13]; ii) syphilis prevalence was higher among FSWs over 30 years and among single and declared or brothel-based FSWs; iii) syphilis was associated with HIV infection.

In our study, the prevalence of syphilis among FSWs and their clients was 2.2%, which is about two times the prevalence of syphilis among women attending antenatal clinics in Togo (1.2% in 2010) [15]. This prevalence is slightly higher than that reported in Northern Sudan (1.5%), and lower than the 2.7% reported in Tunisia [13]. However, these estimates from African countries are low compared to data from China 18.4% [12] and in India (21.1%) among comparable populations [11]. This disparity could be explained by methods used in these studies but also by the impact of the level of social tolerance of sexual diversity as well as by the level of stigma and discrimination against FSWs in some countries. In Togo, the sexual diversity is not well tolerated among the society [19].

The high prevalence of syphilis reflects the extent of risky sexual behaviours such as multiple sexual partnerships and the inconsistent condoms use among this key population. For example, 88% reported having used a condom during their last sexual intercourse before the survey. In a survey conducted in Rwanda, 74% of FSWs reported to have used a condom during their last sexual intercourse [20]. In another study conducted in Uganda, 94% of FSWs reported to have used a condom during the last month of work, among which 45% used the condom systematically [21].

This study shows that age, sex work sites and marital status are associated with the prevalence of syphilis among FSWs. FSWs aged 30 and over were 5.95 times more likely to be infected with syphilis. This result could be partly explained by the relaxation of preventive behaviours that occur among individuals as their age increases. Also, over the course of their lives, older FSWs would have had more opportunity to be exposed to syphilis. Similar results were reported by studies conducted among African and Chinese FSWs in Italy [22, 23]. Unlike our results, Medhi et al. in India, found that young age was a great vulnerability to STIs including syphilis among FSWs and their clients [11].

The results of our study showed that having syphilis was associated with HIV infection. Syphilis in the primary stage is an ulcerative STI It is well known that ulcerative STIs multiply the risk of being infected with HIV by 3 to 5 times [24]. It is important for national HIV and STIs control programs to strengthen STIs surveillance, prevention and management.

### Limitation

This study had limitations related to the methods used. FSWs are difficult to locate due to stigma as well as their illegal status in Togo. We therefore used convenient sampling methods to select the participants and conduct the study during nights mainly. Also, a previous map of sex workers was used to identify brothels and hotspots to conduct this survey. We have therefore partnered with a non-governmental organization that used to work with FSWs in the frame of controlling HIV among key population groups. Another limitation is related to definition of syphilis used in our study where a participant was syphilis positive if the rapid test and TPHA were simultaneously positive. This classification did not allow us to make a difference between a new case and an old case of syphilis.

Another limitation was that data collection was based on face-to-face administrated questionnaires which could be subject to information bias arising from concealment of information.

## Conclusion

The prevalence of syphilis among FSWs and their clients is higher than in women attending antenatal consultations in Togo. The prevalence of syphilis among FSW was associated with age, sex work sites and marital status. Moreover, those who were infected with syphilis were 3.4 times more likely to be infected with HIV. It is therefore important to strengthen interventions which aim at adopting safer sexual behaviour and adequate management of STIs to reduce HIV seroprevalence among FSWs and their clients.

## Abbreviations

ELISA: Enzyme-linked immunosorbent assay
FSW: Female sex worker
OR: Odd ratio
STI: Sexually transmitted infection
TPHA: Treponema pallidum hemagglutinations assay
UNAIDS: Joint United Nations Programme on HIV and AIDS
WHO: World Health Organization
